# A case of microcystic meningioma associated with acute subdural hematoma in the posterior cranial fossa^☆^

**DOI:** 10.1016/j.radcr.2022.06.085

**Published:** 2022-07-31

**Authors:** Kiyotaka Kuroda, Joji Tokugawa, Motoki Yamataka, Kazuki Nishioka, Tetsuya Ueda, Takumi Mitsuhashi, Takashi Mitsuhashi, Makoto Hishii

**Affiliations:** Department of Neurosurgery, Juntendo University Nerima Hospital, 3-1-10 Takanodai, Nerima-ku, Tokyo 177-8521, Japan

**Keywords:** Brain neoplasm, Meningioma, Intracranial hemorrhage, Posterior cranial fossa, Surgery, Pathology

## Abstract

A 53-year-old woman was brought to the emergency room with headache and progressive deterioration of consciousness. Radiological examinations revealed acute subdural hematoma extending along the cerebellar tentorium to the falx cerebri, and a mass lesion with hemorrhage in the left cerebellum, with acute hydrocephalus. Emergency tumor and hematoma removal with decompressive craniectomy of the occiput was performed. Histopathological diagnosis was microcystic meningioma. Postoperatively, the patient recovered to clear consciousness with sequelae of left cerebellar ataxia, cerebellar dysarthria, and vertigo. This case of tentorial microcystic meningioma associated with acute subdural hematoma in the posterior cranial fossa is extremely rare, with only reported 4 similar cases.

## Introduction

Meningioma is one of the most common benign brain tumors and is characterized by abundant vascularity [1], [2], [3], [4], [5]. Intracranial hemorrhage tends to occur with malignant brain tumors such as glioblastomas or metastatic tumors, but is relatively rare in meningiomas [1,2,4,5]. Subarachnoid hemorrhage is the most common type of hemorrhage, whereas acute subdural hematoma is unusual [1,2,4,5]. We present a unique case of microcystic meningioma associated with acute subdural hematoma in the posterior fossa.

## Case description

**Fig. 1:**
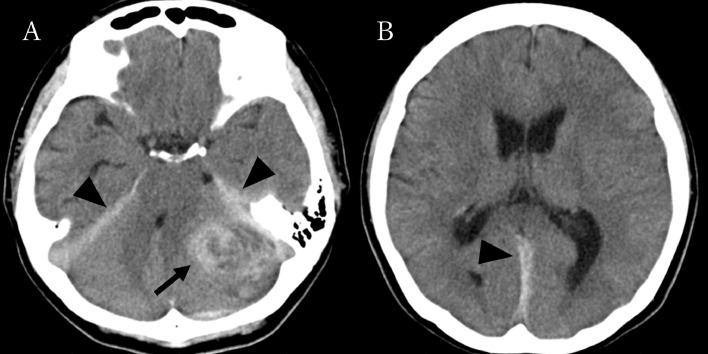
Precontrast computed tomography scans of the head showing (A) a hemorrhagic mass lesion in the left cerebellar hemisphere (arrow) and acute subdural hematoma extending along the cerebellar tentorium (arrowheads), and (B) slightly enlarged ventricles with acute subdural hematoma extending along the falx cerebri (arrowhead).

**Fig. 2:**
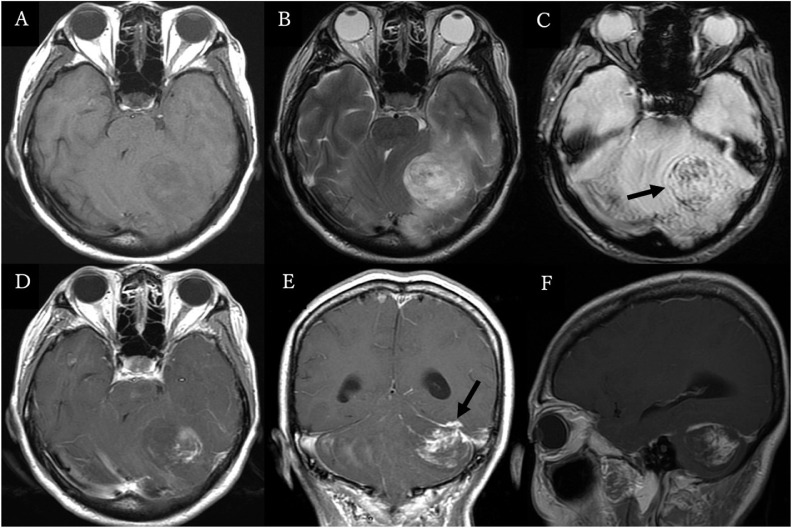
Precontrast magnetic resonance (MR) images of the head showing hypointensity on the T1-weighted image (A), hyperintensity on the T2-weighted image (B), and intratumoral bleeding on the heavy T2-weighted image (C, arrow). Postcontrast MR images showing heterogeneous enhancement of the mass attached to the left cerebellar tentorium (D-F). Note that the supratentorial extension of the tumor was clearly demonstrated on E (arrow).

**Fig. 3:**
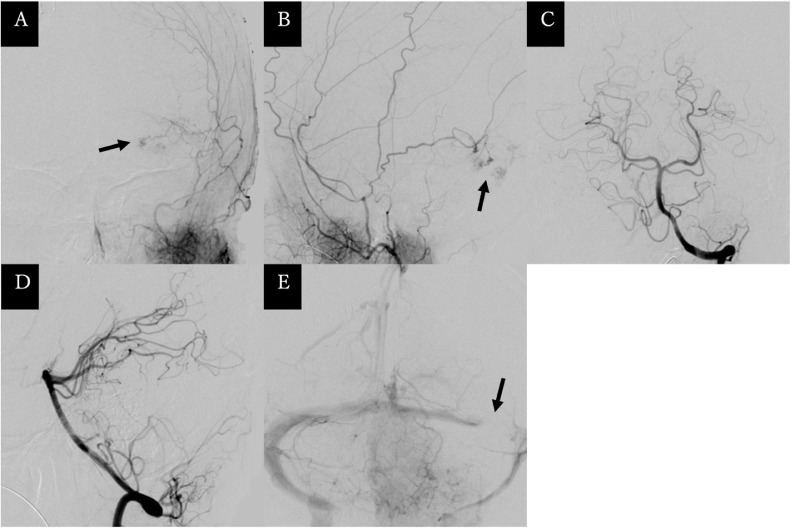
Digital subtraction angiograms of the left external carotid artery, anteroposterior view (A) and lateral view (B), showing partial tumor staining from the left middle meningeal artery (arrows). Digital subtraction angiograms of the left vertebral artery, anteroposterior view (C) and lateral view (D), showing no feeding artery and occlusion of the left transverse sinus (E, arrow).

A 53-year-old woman with no previous medical history was brought to the emergency room with headache and progressive deterioration of consciousness. On arrival, her consciousness was lethargic (Glasgow Coma Scale: E2V2M5 = 9 points). Her pupils were isocoric, but the left eye was adducted, which indicated left abducens nerve paralysis. No paralysis of the extremities was observed. Head computed tomography revealed acute subdural hematoma extending along the cerebellar tentorium to the falx cerebri and a hemorrhagic mass lesion of 4 cm in maximum diameter in the left cerebellar hemisphere. The cerebral aqueduct was deviated to the right, resulting in acute hydrocephalus (Fig. 1). Precontrast magnetic resonance (MR) imaging of the head revealed a mass lesion with hemorrhage, which appeared as hypointensity on T1-weighted images and hyperintensity on T2-weighted images. Postcontrast MR imaging showed that the lesion was attached to and partially extended above the cerebellar tentorium with heterogeneous contrast effect in the mass lesion and a “dural tail sign” along the tentorium (Fig. 2). Cerebral angiography demonstrated that part of the mass was fed by the left petrosquamous branch from the left middle meningeal artery, but not by the vertebral artery. The left transverse sinus was occluded in the distal third (Fig. 3). The differential diagnosis included meningioma, solitary fibrous tumor/hemangiopericytoma, metastatic brain tumor, and malignant glioma.

**Table 1:** – Summary of previous cases of the posterior cranial fossa meningioma presented with acute subdural hematoma [3].

Author (year)	Age (y), sex	Symptoms	Hemorrhage type	Location	Histology	Outcome
Yasagil et al. (1976)	50, F	Headache, Consciousness disturbance, Hemiparesis	SDH, ITH, SAH	CP angle	Transitional meningioma	Morbidity
Martinez-Large et al. (1991)	67, F	Headache, Vomiting	SDH, SAH	Petrous bone	Transitional meningioma	Dead
Bosniak et al. (2005)	44, F	Headache, Vomiting, Vertigo	SDH, ITH, SAH	Petrotentorial	Atypical meningioma	Normal
Mitsuhara et al. (2006)	60, F	Headache, Gait disturbance	SDH, ITH, SAH	Petrotentorial	Meningothelial meningioma	Normal
Our Case	53, F	Headache, Consciousness disturbance	SDH, ITH, SAH	Tentorial	Microcystic meningioma	Morbidity

CP, cerebellopontine; F, female; ITH, intratumoral hemorrhage; SAH, subarachnoid hemorrhage; SDH, subdural hemorrhage.

**Fig. 4:**
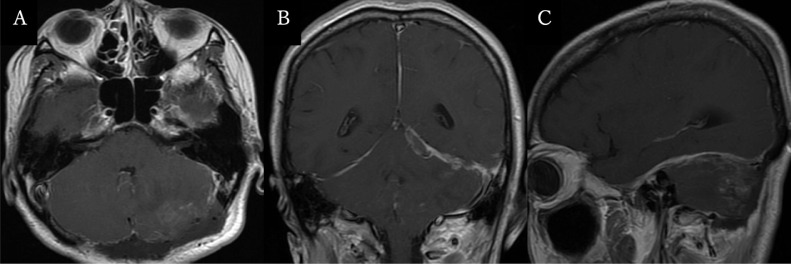
Postoperative magnetic resonance images, axial (A), coronal (B), and sagittal views (C), showing the tumor and hematoma have been largely removed.

**Fig. 5:**
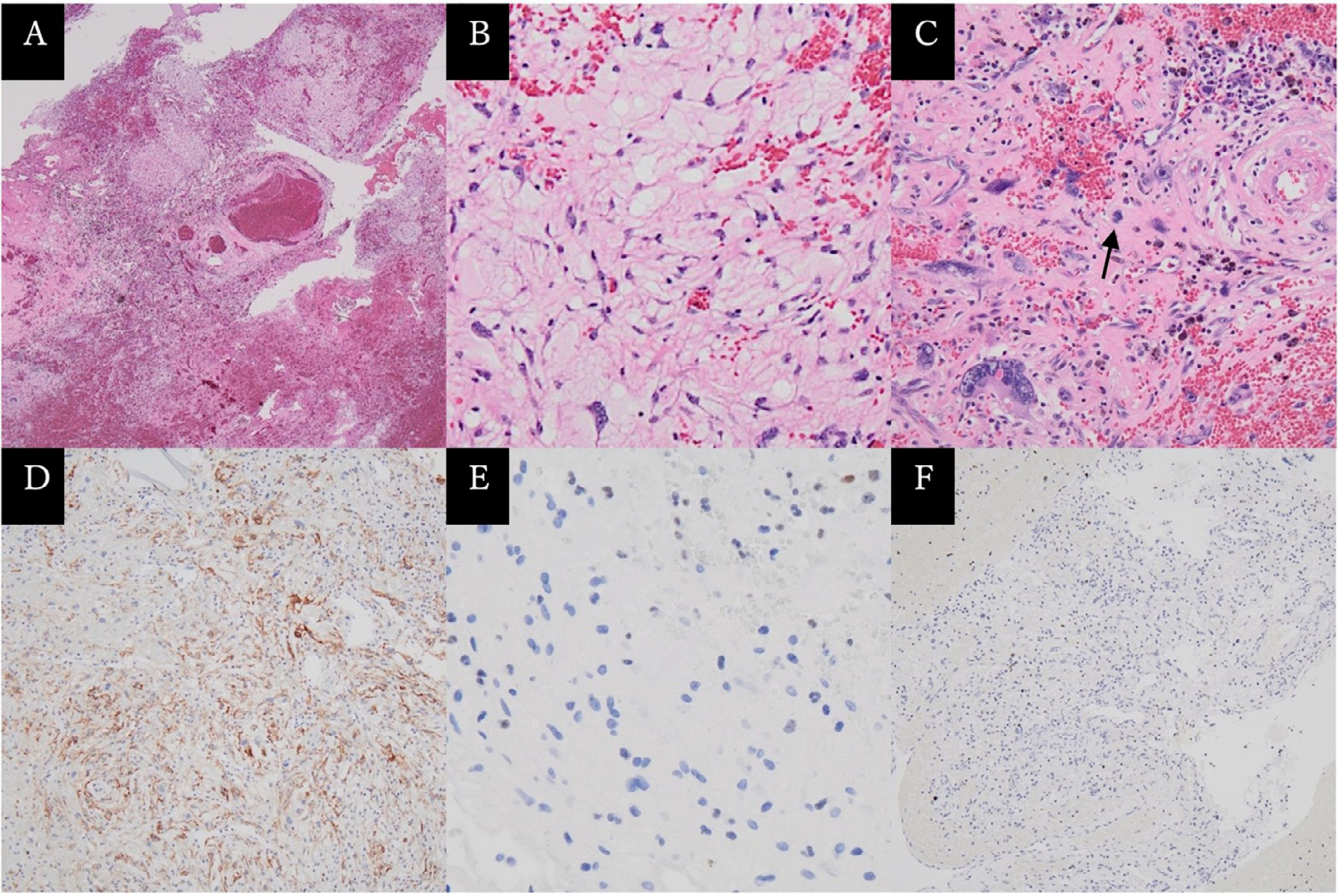
Photomicrographs of the surgical specimens with hematoxylin-eosin staining showing tumor cell proliferation against a background of bleeding components (A), microcystic components (B), and nuclear atypia (C, arrow). Immunohistochemical staining showing positive epithelial membrane antigen staining (D), negative glial fibrillary acidic protein staining (E), and Ki-67 index of <1% (F).

Emergency tumor and hematoma removal with decompressive craniectomy of the occiput was performed. The tumor was extramedullary and attached to the tentorium. The surgery was completed leaving only slight residual supratentorial lesion (Fig. 4). Postoperative histopathological examination with hematoxylin-eosin staining revealed proliferation of tumor cells with hemorrhagic component and nuclear atypia with multiple microcysts. Immunohistochemical staining showed positive for epithelial membrane antigen staining, negative for glial fibrillary acidic protein staining, and Ki-67 index of <1% (Fig. 5). The diagnosis of microcystic meningioma was made based on preoperative imaging, intraoperative findings, and histopathological findings. After postoperative neurorehabilitation, the patient recovered to clear consciousness with sequelae of left cerebellar ataxia, left abducens nerve paralysis, cerebellar dysarthria, and vertigo.

## Discussion

Intracranial hemorrhage due to brain tumor accounts for 0.9%-10.2% of all cases of intracranial hemorrhage [5]. Malignant gliomas and metastatic brain tumors are the most likely to cause intracranial hemorrhage, and meningiomas cause as few as 1.3%-2.4% of all cases [1,2,5]. Subarachnoid hemorrhage is the most common type of hemorrhage associated with meningioma, whereas subdural hematoma is rare, with 57 cases reported [1,4]. Among these 57 cases, the ratio of males to females was approximately 1:2, the most common location was the cerebral convexity (63%), and the most common pathological subtypes were meningothelial (43%) and angiomatous meningioma (9%) [1]. Subdural hematoma caused by meningioma in the posterior fossa is extremely rare, with only 4 cases reported (Table 1). Microcystic meningioma is a rare subtype of meningioma, accounting for only 1.6% of all cases [6], [7], [8]. Although the contrast patterns may vary with subtypes, meningiomas are usually demonstrated as homogeneously enhancing masses in postcontrast MR imaging, and show remarkable tumor blush in angiography because of their hypervascularity [9,10]. The contrast pattern of microcystic meningioma is sometimes described as “faint reticular pattern,” which represents a spider's web-like enhancement inside the mass [7,8]. Histopathologically, microcystic meningioma consists of sheets of spindle tumor cells with a loose background and cystic structure with stellate cells [7,11]. The mixture of these 2 different components attribute to the “faint reticular pattern” on postcontrast MR imaging. Even though the “faint reticular pattern” is characteristic of microcystic meningioma, meningeal Ewing sarcoma/peripheral primitive neuroectodermal tumor sometimes shows a similar image and is difficult to differentiate [11]. Two rare characteristics, a rare bleeding type and a rare meningioma subtype, of the present unique case made the preoperative diagnosis difficult.

Intracranial hemorrhage with meningioma has various causes, such as 1) hemorrhage due to failure of the feeding blood vessels due to hypertrophy and dilation of the blood vessel walls as the tumor grows, 2) hemorrhage due to failure of abnormal blood vessels in the tumor, 3) hemorrhage due to compression and occlusion of the veins and venous sinuses around the tumor as the tumor grows, and 4) hemorrhage due to trauma [1]. In this case, 1), 2), and 4) are unlikely based on the medical history and preoperative findings. The intraoperative findings revealed no obvious draining vein that could cause hemorrhage, but preoperative cerebral angiography showed occlusion of the left transverse sinus, so the hemorrhage could be due to congestion caused by occlusion of the vein leading out of the tumor, which corresponds to cause 3).

## Conclusion

We describe an unprecedented case of tentorial microcystic meningioma associated with acute subdural hematoma of the posterior cranial fossa. The preoperative differential diagnosis was difficult because of the atypical preoperative imaging findings of the meningioma.
